# Graph modelling for tracking the COVID-19 pandemic spread

**DOI:** 10.1016/j.idm.2020.12.002

**Published:** 2020-12-13

**Authors:** Rasim Alguliyev, Ramiz Aliguliyev, Farhad Yusifov

**Affiliations:** Institute of Information Technology, Azerbaijan National Academy of Sciences, Baku, Azerbaijan

**Keywords:** COVID-19, Pandemic, Modelling, Epidemiological characteristics, Graph visualization

## Abstract

The modelling is widely used in determining the best strategies for the mitigation of the impact of infectious diseases. Currently, the modelling of a complex system such as the spread of COVID-19 infection is among the topical issues. The aim of this article is graph-based modelling of the COVID-19 infection spread. The article investigates the studies related to the modelling of COVID-19 pandemic and analyses the factors affecting the spread of the disease and its main characteristics. We propose a conceptual model of COVID-19 epidemic by considering the social distance, the duration of contact with an infected person and their location-based demographic characteristics. Based on the hypothetical scenario of the spread of the virus, a graph model of the process are developed starting from the first confirmed infection case to human-to-human transmission of the virus and visualized by considering the epidemiological characteristics of COVID-19. The application of graph for the pandemic modelling allows for considering multiple factors affecting the epidemiological process and conducting numerical experiments. The advantage of this approach is justified with the fact that it enables the reverse analysis the spread as a result of the dynamic record of detected cases of the infection in the model. This approach allows for to determining undetected cases of infection based on the social distance and duration of contact and eliminating the uncertainty significantly. Note that social, economic, demographic factors, the population density, mental values and etc. affect the increase in number of cases of infection and hence, the research was not able to consider all factors. In future research will analyze multiple factors impacting the number of infections and their use in the models will be considered.

## Introduction

The current socio-economic and political processes, globalization, rising number of conflicts, migration problem, environmental pollution, an increase in infectious diseases and epidemics affect everyday life of individuals significantly and impedes the sustainable development of countries. Currently, the problem of the spread of infectious diseases is at the focus of the world community and plays a critical role in decision making in the public health sector. As of 28 October 2020, the rapid spread of the novel coronavirus disease (COVID-19) has caused the infection of approximately 43.8 million people and accounts for more than 1.1 million deaths (WHO, 2020). COVID-19 virus poses a serious threat for the human health, social life and development, production and international relations. In this regard, forecasting is an important issue to prevent the spread of the disease and carry out preventive measures by considering the epidemiological characteristics of COVID-19.

Modelling is one of the broadly used tools for the forecasting related to the epidemic situation in the society. At present, it is a topical problem to develop imitation models of complex systems such as the process of spread of an infection. The existence of adequate mathematical model is paramount for obtaining accurate forecast regarding the level of the spread of a disease and studying the process (Currie et al., 2020; Michaud et al., 2020). The modelling of pandemic processes can be viewed as an integral component of electronic medical demographics system of the government and play a prominent role in prediction and effective decision making.

Planning and forecasting tasks are important for mitigating the sudden and potentially catastrophic impact of the infectious disease pandemic on the society; however, it is not an easy task. During a pandemic, decisions are made with limited experience in a rapidly changing and uncertain situation which we observe the above described in the novel coronavirus pandemic. The historically occurring pandemics have caused the death of 10 million people all over the world (Shearer et al., 2020). Although, currently, the existence of vaccines against some infectious diseases is reassuring, the cities and countries connected via air transportation facilitate the rapid transmission of viruses such as COVID-19. In particular, large number of individuals spend a relatively long period of incubation and hence, can transmit the disease to other people without knowing for 10–14 days when no symptoms are manifested (ECDC Technical Report, 2020). In this regard, models are important tools for planning the pandemic and carrying out response measures (Currie et al., 2020; Shearer et al., 2020). Certainly, it is not possible to forecast the location or time of occurrence of next pandemic; however, models preserve large potential for increasing the effectiveness of response measures once pandemic occurs (Currie et al., 2020; Holmes et al., 2018; Kerkhove & Ferguson, 2012).

Modelling is widely utilized by all governments and World Health Organization (WHO) for determining the best strategies in order to mitigate the impact of COVID-19 (Riggins, 2020; Currie et al., 2020). These are mainly epidemiological models oriented to studying the spread of the disease and impact of various interventions. However, global pandemic is not only related to the transmission of diseases and causes the emergence of several problems, and each of them requires a different model for obtaining the best solution (Riggins, 2020; Kristiansen, 2020). The aim of the study is to support decision making for the purpose of modelling of the spread of COVID-19 pandemic, forecasting of the process and eliminating the consequences. Stochastic, time series analysis, agent-based, autoregression, hybrid and etc. models are applied in the literature (Ivorra et al., 2020; Riggins, 2020; Kristiansen, 2020; Michaud et al., 2020; Sameni, 2020). The use of geographical information systems and big data technologies plays an important role in fast aggregation of big data from multiple sources, data visualization, spatial tracking of infected, prediction of transmission, spatial segmentation of epidemic risk, support for decision making and the evaluation of the effectiveness of preventive measures from different aspects in fighting the pandemic (Zhou et al., 2020).

The main aim of this article is to develop an adapted conceptual model that allows for modelling the transmission process by considering specific characteristics of COVID-19 which is different from existing SIR, SEIR (Michaud et al., 2020; Ivorra et al., 2020; Sameni, 2020) or other general-purpose epidemic models. Different scenarios of the model will enable the assessment and forecast of detected and undetected cases of infection and death by considering the dynamics, time and location-based demographic characteristics (age, nationality, gender, profession, medical condition and etc.) starting from the moment of registration of the first infection case.

## Modelling of COVID-19 pandemic: a literature review

In the current study related works, we observe some mathematical models attempting to describe the dynamics of COVID-19 evolution in literature (Khan & Atangana, 2020; Ivorra et al., 2020; Roosa et al., 2020; Kucharski et al., 2020). Roosa et al. (2020) presents a phenomenological model applied to the spread of other diseases different from COVID-19 and attempts to forecast and asses the cumulative number of detected cases for short term. In another study Kucharski et al. (2020) propose models such as SEIR with smaller variations and some stochastic components.

Khan and Atangana (2020) review the mathematical modelling and dynamics of new coronavirus in their study. The study elaborates the short details of interaction first between bats and unknown hosts and thereafter between humans and seafood market called the reservoir of infection. According to researchers, as bats or their hosts (can be other wild animals) transmit the infection and are direct contacts, the seafood market is considered to be the primary source of infection. The proposed model is developed with an assumption that the seafood market is a sufficient source of infection for infecting humans. The mathematical results of the model are presented, and fractional model is developed. Numerical calculations are carried out that could be useful for minimizing the transmission and several graphical results are presented.

Ivorra et al. (2020) study is dedicated to the mathematical modelling of the spread of novel coronavirus disease by considering detected cases of infection. They propose a new θ-SEIHRD model different from existing SIR, SEIR models for the modelling of the spread of new coronavirus disease. The main advantage of the model is that it takes into consideration known features of the disease including undetected cases of infection, sanitary conditions and state of infection of hospitalized individuals. Authors study China’s experience as the country spreading the virus (including Chinese Mainland, Macao, Hong Kong and Taiwan as stated by WHO COVID-19 reports); the general situation within the country is investigated and the parameters of the model are determined by using reported information. It is noted that the parameters used in the model might be of interest for the assessment of spread of COVID-19 in other countries (Ivorra et al., 2020).

Pavlyshenko (2020) investigates various regression approaches for the modelling of the spread of COVID-19 and its impact on stock market. The forecast of the spread of coronavirus is studied with the application of logistic curve and Bayesian regression. The impact of COVID-19 is studied with regression approach and its impact is compared with the impact of other crises. The opportunity of the quantitative measurement of uncertainty in Bayesian regression can be useful information for experts while choosing models (Pavlyshenko, 2020). Lin et al. (2020) proposes a conceptual model of COVID-19 epidemic in Wuhan by considering individual behavioral reactions and government actions, for example, the extension of leave, travel restriction, hospitalization and quarantine measures. The model has a simple structure, it can successfully predict the progress of COVID-19 epidemic and thus allows for understanding the spread trends (Lin et al., 2020). Li et al. (2020) focus on the analysis and prediction of the spread of COVID-19 pandemic. The analysis of existing data characterizing the epidemiological situation in Hubei shows that the error produced by the model is sufficiently small in comparison with official data. The study investigates the factors affecting the spread of COVID-19, for instance, the number of recovered individuals, the period of incubation and the average number of days of treatment.

Kucharski et al. (2020) investigates the mathematical modelling of early dynamics of the spread and control of COVID-19 pandemic. The dynamic model of stochastic transmission utilizes the data of numerous open datasets related to the cases of infection occurring in Wuhan and exported abroad to assess the dynamics of early spread. The authors attempted to approximate how the spread has changed between December 2019 and February 2020 in Wuhan and outside its borders by combining four datasets and using the mathematical model of COVID-19 spread. Another study by Yang and Wang (2020) proposed a mathematical model to investigate the current state of spread of novel coronavirus disease emerged in Wuhan in 2019. The model characterizes multiple ways of transmission in the dynamics of infection and emphasizes the role of environment reservoir in the transmission of this disease. Moreover, the model utilizes non-constant transmission rates changing according to the epidemiological situation and environmental conditions and reflecting the impact of fight measures against diseases. Obtained analytical and experimental findings increase the likelihood of the coronavirus infection becoming an endemic infection and emphasizes the need for long-term prevention measures for the disease prevention in this case (Yang & Wang, 2020). Imai et al. (2020) reviews the mathematical modelling of potential epidemic trajectory in order to determine the scale of the disease spread in Wuhan by considering human-to-human transmission of the virus. It is shown that the measures carried out in fighting the virus must prevent 60% of transmission in order to keep the spread under control (Imai et al., 2020).

Zhao et al. (2014) investigates the multiple routes transmitted epidemic process on multiplex networks. The authors propose detailed theoretical analysis to accurately calculate the epidemic threshold and outbreak size. The main outcomes of the study that the epidemic can spread across the multiplex network even if all the network layers are well below their respective epidemic thresholds. Noted that a strong positive degree–degree correlation of nodes in multiplex network could lead to a much lower epidemic threshold and a relatively smaller outbreak size.

Chen et al. (2020) proposed Bats-Hosts-Reservoir-People network model for the modelling of transmission potential from the source of infection (bats as assumed) to human. However, as the investigation of Bats-Hosts-Reservoir-People network is complex and public opinion is inclined towards the assumption that the virus is transmitted from seafood market (Huanan Seafood Wholesale Market) to human, the model is simplified and Reservoir-People (RP) transmission network is studied. Research results show that, compared to other severe respiratory syndromes, the transmission potential of COVID-19 is higher than Middle East Respiratory Syndrome (MERS), similar to severe acute respiratory syndrome (SARS), but lower than MERS syndrome observed in the Republic of Korea (Chen et al., 2020).

Zhao et al. (2020) the study is dedicated to estimating the unreported number of novel Coronavirus (2019-nCoV) cases in China in the first half of January 2020On the based of the proposed approach modelled the epidemic curve of 2019-nCoV cases, in mainland China from 1 December 2019 to 24 January 2020 through the exponential growth. The number of unreported cases was determined by the maximum likelihood estimation. The author confirmed that the initial growth phase followed an exponential growth pattern. As a result, noted that the reporting rate after 17 January 2020 was likely to have increased compared with the situation from 1 to 17 January 2020 on average, and it should be considered in future investigation.

Giordano et al. (2020) reviewed the modelling of COVID-19 epidemic and the implementation of measures to fight the virus at the scale of population of Italy. In order to assist the effective planning of fight strategy, a new model forecasting the progress of the pandemic has been proposed. The model considers 8 stages of the infection. These are susceptible (S), infected (I), diagnosed (D), ailing (A), recognized (R), threatened (T), healed (H), and extinct (E), stages collectively termed as SIDARTHE (Giordano et al., 2020). Obtained findings show the need for the implementation of social distancing measures alongside the increase in the number of tests and contact tracing measures for the elimination of continuing COVID-19 pandemic. The proposed approach enables the forecasting of possible scenarios of the implementation of counter measures.

Kissler et al. (2020) focus on projecting the transmission dynamics of SARS-CoV-2 through the postpandemic period. The study used estimates of seasonality, immunity, and cross-immunity for human coronavirus OC43 (HCoV-OC43) and HCoV-HKU1 using time-series data from the United States to inform a model of SARS-CoV-2 transmission. The authors projected that recurrent wintertime outbreaks of SARS-CoV-2 will probably occur after the initial, most severe pandemic wave. The study used existing data to build a deterministic model of multiyear interactions between existing coronaviruses, with a focus on the United States, and used this to project the potential epidemic dynamics. The author reckons that the long-term dynamics of SARS-CoV-2 strongly depends on immune responses and immune cross-reactions between the coronaviruses, as well as the timing of introduction of the new virus into a population.

The majority of proposed models emphasize the prominent role of the direct transmission from human to human in COVID-19 epidemic (Chan et al., 2020; Sahin et al., 2020; Yang & Wang, 2020). On the other hand, despite the existence of a large number of proposed models, the majority does not take into consideration the epidemiological characteristics of COVID-19 such as social distance, the contact duration with infected person as well as the age, gender, nationality, profession, the presence of chronic diseases which affect the scale of spread. For this purpose, the article proposes a conceptual model of COVID-19 epidemic by considering social distance, contact duration with the infected person and information about each individual (age, gender, nationality, profession, the presence of chronic diseases etc.).

## Factor affecting the spread of COVID-19 and main characteristics

The modelling of relocation of urban population expresses the dynamic nature of social network and an infectious agent is spread among population by using analogous chain of relations. In this regard, the model must be able to reflect the evolution of an infection in real life accurately to ensure the reliability of forecasts (Ketchell, 2020). As several diseases such as flu have been studied for decades, the development of a model based on statistical data enables more realistic forecasts in this case. The models of flu infection are used for decision making regarding vaccine formulas and other seasonal flu medications every year. In contrast, researchers possess few information about novel coronavirus disease and hence, the modelling of the spread of existing COVID-19 virus is a complex issue. At present, models in some studies utilize flu information assuming that COVID-19 virus behaves as flu. Other approaches assume that novel coronavirus behaves like SARS-CoV virus that caused SARS epidemic in 2003 (Ketchell, 2020). Other models may propose different assumptions regarding COVID-19; however, all of them attempt to obtain the required information. It is understandable that this information does not exist just yet. In this regard, the proposition of different assumption is considered to cause the development of various forecasts (Chen et al., 2020; Gleick, 2020; Ketchell, 2020).

On the other hand, researchers utilize models in order to assess important epidemiological characteristics of the disease such as the impact of public health interventions including the period of incubation, ability to infect, asymptomaticity and severity as well as social distancing, medical examination at airports, travel restrictions and contact tracing (Michaud et al., 2020). It is to be noted that existing models can be considered as an important tool for understanding the disease, implementing response measures and political decisions; however, at the same time, this leads to disagreements as their approaches and proposed assumptions differ from each other significantly (Gleick, 2020; Holmdahl & Buckee, 2020).

**Fig. 1:**
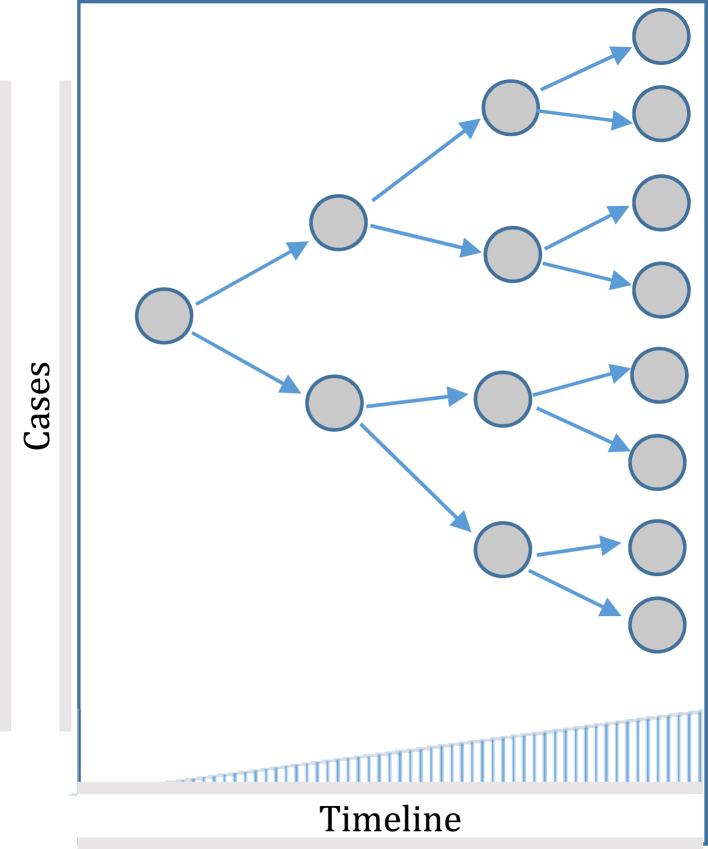
Process of spread of infection.

In general, most people are vulnerable to the infection at early stages of epidemic and the spread of this disease from human to human can be modelled as a stochastic “branching process”. As shown in the graph, if an infected person on average infects two individuals, the number of infected individuals doubles at each stage and this process grows exponentially. In Fig. 1 graphically describes the process of the spread of virus.

It is evident that an infected person does not necessarily infect others. Several factors affect the probability of being infected. In case of pandemic, the speed of infection depends on average number of people one person can infect and the time needed for these people to become contagious.

Hence, it is important to determine the main dynamics of transmission for accurate prediction of COVID-19. According to WHO, following factors affect the spread of COVID-19 (WHO IHR, 2020):

⁃ How many individuals does one person infect on average? (According to WHO information, “reproduction number” is considered to be 1.4–2.5 currently)
⁃ The period of infection of a person in the same environment with the infected? (15 min or more and less than 2 m distance (ECDC Technical Report, 2020))
⁃ What is the period after infection before the symptoms start to be manifested? (“incubation period” is assumed to be approximately 5.1 days)
⁃ What is the ratio of spread before symptoms start (if present)?

The collection of preliminary data enables precise predictions regarding COVID-19 progress in order to take into consideration mentioned factors and known specific characteristics in the model.

Currently, several applications are being used in China, South Korea, Singapore, Israel and other countries to monitor social distancing among preventive measures. Considering that practically everyone owns smartphones, it is possible to detect the location of these devices using geolocation systems. Based on the data mining methods, a warning signal can be sent regarding the maintenance of social distance and undesirable approach distance when needed by monitoring the distance between individuals in densely populated areas. In particular, it is possible to determine risk groups and maintain strict monitoring between them. At present, contact tracing applications are used by governments in several countries (Panzarino, 2020; TraceTogether; Stopp Corona APP; QR health code; eRouška; Morrow (2020); COVID Community Alert). Among those, Stopp Corona (Austria), Alipay Health Code (Chine), eRouška (Czechia), StopCovid (France), COVID Community Alert (Italy), TraceTogether (Singapore), COVID-19 Apple/Google App (US) and etc. can be mentioned. Companies such as Google and Apple state that it is possible to apply contact tracing systems based on joint Bluetooth Low Energy technology and privacy-preserving cryptography (Panzarino, 2020; Apple & Google partner on COVID-19). For instance, TraceTogether app is used in Singapore in order to obtain information via smartphone regarding possible contact with patients infected with coronavirus (TraceTogether). According to criterion of the ministry of health of Singapore, a “close contact” implies a distance less than 2 m for 30 min. The ministry of health of Israel checks the presence of risk of infection with coronavirus in case of being in proximity of or in contact with a virus host with “HaMagen” app developed for mobile phones (HaMagen). Two different methods are applied in order to measure the distance between individuals via smartphones. One of those is the use of geolocation information utilizing GPS (Global Positioning System) or GNSS (Global Navigation Satellite System) systems – in this case, the positioning accuracy is 0.6 m and can reach 10 cm with the time (Connell, 2015). But the loading of local network or unsustainability and unreliability of the system in indoor locations are considered to be main drawbacks (Connell, 2015; Slamich, 2020). The measurement of distance with Wi-Fi and Bluetooth signals is characterized with large measurement error in close distances. From this point of view, the use of ultra-wideband (UWB) radio signals can significantly increase the measurement accuracy in close distances (5–10 cm) (Connell, 2015). At present, UWB technology is preferred for the development of devices determining the location in specific indoor locations. The utilization of this technology will play in important role in measuring social distance and determining the risk of infection.

## Modelling of the process COVID-19 spread

The rapid spread of novel coronavirus disease has attracted the world community’s attention due to its serious threat to human health. In this regard, modelling is paramount for the mitigation of the catastrophic impact of infectious diseases and pandemics on the society; however, that is not an easy task. COVID-19 is a disease caused by a novel virus resulting in an emergency situation all over the world. Hence, there is a need for developing a model which takes into account specific known characteristics of the disease distinct from traditional models in order to analyze the process of the spread of COVID-19 and forecasting its dynamics. Considering the approaches proposed in the literature, we presume that each individual is attributed to one of the following groups according to the known characteristics of COVID-19 pandemic (Ivorra et al., 2020; Wang et al., 2020):

**Table:** 

Compartments	Description	Denotation
First confirmed case	First patient infected with COVID-19	*P*
Exposed	The individual has been close contact with infected person. The individual is in the incubation period after being infected by the disease pathogen and has no visible clinical signs yet.	*E*
Infectious	The individual has finished the incubation period, may infect other people and starts developing clinical signs.	*I*
Infectious undetected	The individual can still infect other people, have clinical signs but is not be detected and registered by authorities. After this period, an individual in this compartment pass to the Recovered (*R*_*un*_) or the Dead (*D*) compartments depending on disease severity and medical conditions.	*I*_*un*_
Recovered but undetected	The individual was not previously detected as infectious, also maybe the asymptomatic case, recovered and has developed a natural immunity to the virus.	*R*_*un*_
Hospitalized or in quarantine at home and will recover	The individual is in hospital (in isolation, or quarantine at home) and can still infect other people. The individual was detected and reported by the authorities. As a mild case at the end of this state, an individual will recover.	*H*_*R*_
Recovered but previously detected as infectious	The individual was previously detected as infectious, recovered and has developed a natural immunity to the virus.	*R*_*de*_
Hospitalized but will die	The individual is hospitalized and can still infect other people. As a severe case at the end of this state, an individual will die.	*H*_*D*_
Dead	The individual has not survived a novel coronavirus disease.	*D*

**Fig. 2:**
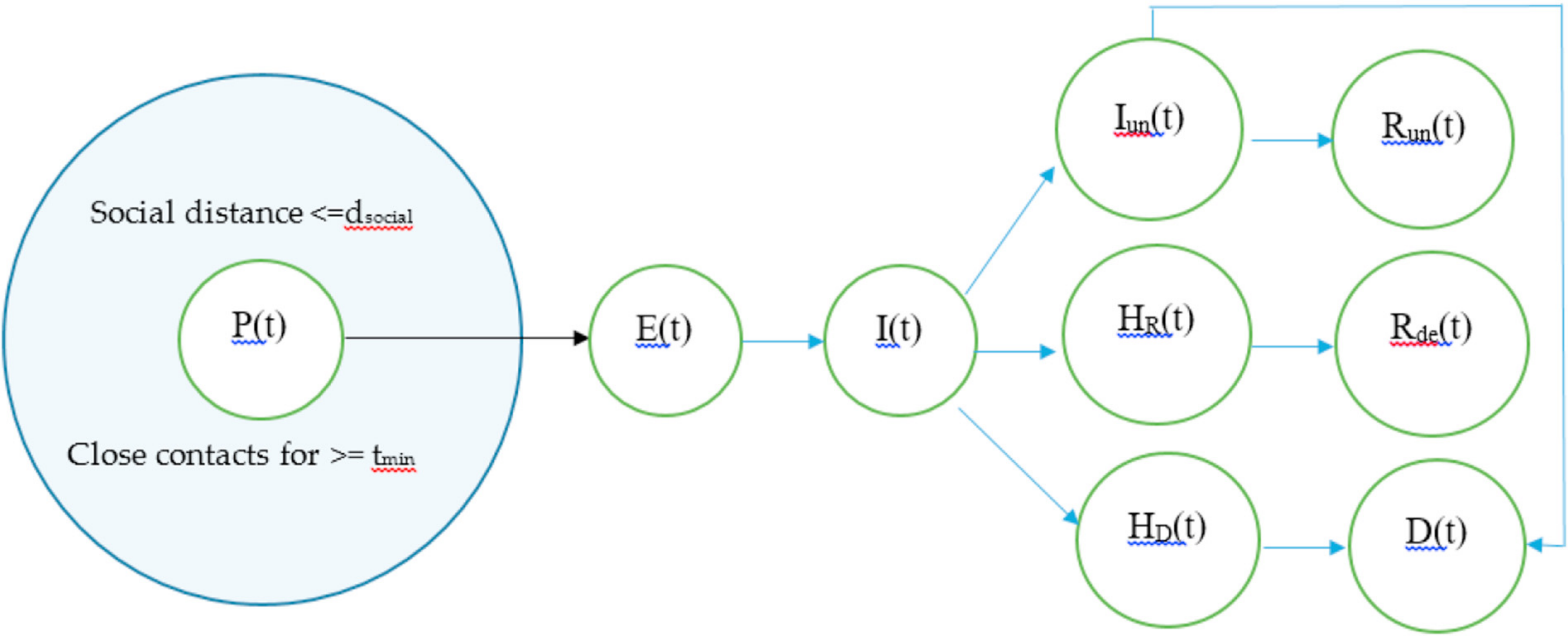
Graphical scheme representing the interactions among different stages of COVID-19 infection in the conceptual model.

The model is utilized for the assessment of the spread dynamics of disease in a specific time period starting from the detection of the first case of infection and development of various forecasts. We can start modelling at t_0_ point of time at any location where the first case of infection (P) was detected. In this case, individuals with the potential to be infected will be attributed to one of E, I, Iun, Run, H_R_, R_de_, H_D_ and D groups in t period of time considering the social distance and minimum contact with an infected person. Takin into account the epidemiological characteristics of COVID-19, the process of the spread of the disease is presented in Fig. 2.

**Fig. 3:**
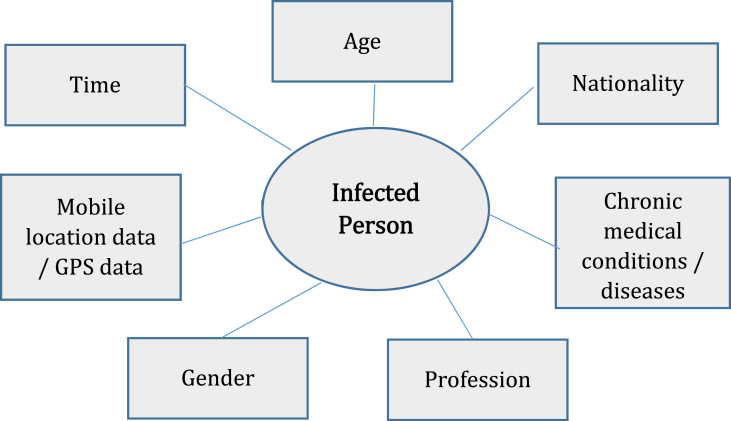
Available information about infected person.

We think that the following personal information for each registered case of infection must be taken into consideration in order to study the dynamics of spread of each pandemic, increase the effectiveness of forecasts and conduct demographic studies (as shown in Fig. 3). This information can be obtained from population registry, mobile operators, e-health system and other government registries.

From this point of view, the use of proactive strategies based on the analysis of large-volume data, Data Science, decision making theory and predictive analytical methods is considered purposeful in order to keep the epidemiological situation stable in the fight against the emerged pandemic as a result of the spread of a virus such as COVID-19 (Zhou et al., 2020). The utilization of geographical information systems and location information plays an important role in determining the spatial spread of the epidemic, preventive and fight measures, resource allocation and decision making at individual, group and regional level.

The presence of electronic medical demographics system will assist the coverage of the majority of health facilities and population in order to facilitate the implementation of preventive measures. This system will enable the collection and processing of information regarding the state of people’s health, the monitoring of the situation and remote diagnostics of the probability of being infected with coronavirus. The modelling of pandemic process and implementation of proactive strategies will be conducive to decreasing the number of infections significantly, localizing the locations where quarantine is implemented and shortening the period of isolation depending on the incubation period.

## COVID-19 pandemic graph-based visualization

The simulation of epidemiological situation allows for testing the efficiency of fight measures on a model in order to prevent the spread of infectious diseases. Despite the presence of various approached to the modelling of the spread of infectious diseases, it is known that an infectious agent is spread via interaction between individuals as in social network. Formally, social networks can be described with graph and in this case, the edges of the graph denote individuals and arrows denote the set of interactions. In this regard, graph models can be utilized while modelling and visualizing the pandemic processes. Graph models play an important role in forecasting the disease spread and supporting decision making. In particular, the use of colored graphs enables the visualization of a pandemic process and can be paramount for forecasting, assessment of the effectiveness of pandemic processes, implementation of proactive processes and decision making. Alongside the hypotheses proposed in existing approaches, uncertainty regarding epidemic characteristics is one of the drawbacks of these models.

**Fig. 4:**
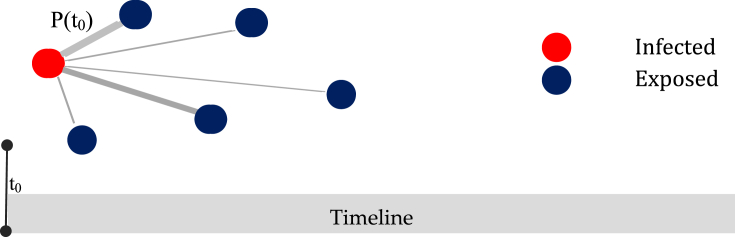
Graph model of virus transmission at t_0_ time point.

The majority of simulation models are developed based on uncertain information without insufficient statistical data. Moreover, only data based on detected cases of infection is utilized in pandemic environment. In this regard, it is a complex issue to model the rapidly spreading disease such as COVID-19 based on limited statistical data in a short period of time and provide accurate forecasts. Similarly, uncertainties regarding the route of spread of the disease and undetected infections imply that the proposed logic is not completely confirmed. The utilization of graph-based models in the modelling of a pandemic can be justified with the fact that it enables the consideration of multiple factors affecting the pandemic process and conduction of numerical experiments. Formally, if we describe social interactions with a graph S=(I,C), I – is the set of edges (individuals), C – is the set of arrows (interactions). It is obvious that each individual is active at a certain position and can change its position with the impact of factors with the time. As shown in Fig. 3, individual characteristics can include age, education, gender, location information, the presence of chronical diseases and etc. The main advantage of the graph model is its stable expansion despite the increase in the number of edges and arrows. Although the datasets of infected individuals with COVID-19 are not accessible due to confidentiality and other objective reasons, it is possible to visualize the human-to-human transmission of the virus experimentally. For this purpose, we can consider the scenario of COVID-19 infection hypothetically. Assume that the first case of infection is recorded at t_0_ time point and the colored graph of individuals in contact with infected person is given in Fig. 4 via Flourish visualization tool.

Considering the epidemiological characteristics of COVID-19, it is possible to visualize the graph model at t_1_ time period by tracking the process of human-to-human transmission of the virus.

**Fig. 5:**
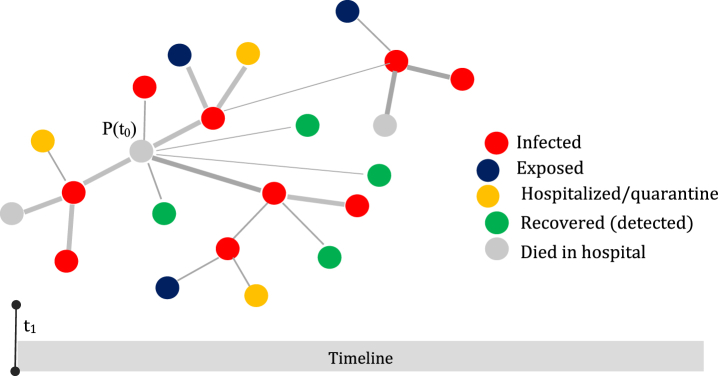
Graph model of virus transmission at t_1_ time point.

As seen from Fig. 5, only confirmed case of infection are included in the graph model.

In the study to define the peak level of the infection at certain t_peak_ time point, we used two indicators such as the average recovery and average infection coefficients. The infection reach peak according to if $t_{peak}=\max_{t}\left\{\frac{\alpha \left(t\right)}{\beta \left(t\right)}\right\}$, where, α(t) – average recovery coefficient, which depends on different factors such as healthcare system condition in country, social, economic factors, mental health impact and etc.; β(t) – average infection coefficients, which depends on cultural-psychological, mental factors, population density and etc. In starting at $t_{0}$ moment $\beta \left(t_{0}\right)=\frac{S\left(t_{0}\right)}{P\left(t_{0}\right)}$ , where, $S\left(t_{0}\right)$ – the number of susceptibles and $P\left(t_{0}\right)$ – population of the country. The infection reach to peak $t_{peak}$ when $\left\{\frac{\alpha \left(t\right)}{\beta \left(t\right)}\right\}$ ratio is maximum in graph model.

**Fig. 6:**
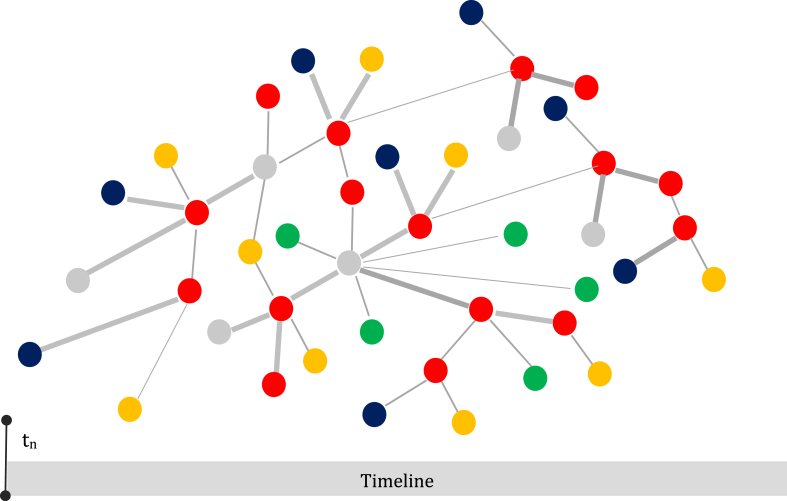
Graph model of virus transmission at t_n_ time point.

As a result of the rapid spread of the virus, it is possible to analyze the spread network of the virus considering that the infection is at its peak at certain tn time point (as shown in Fig. 6).

**Fig. 7:**
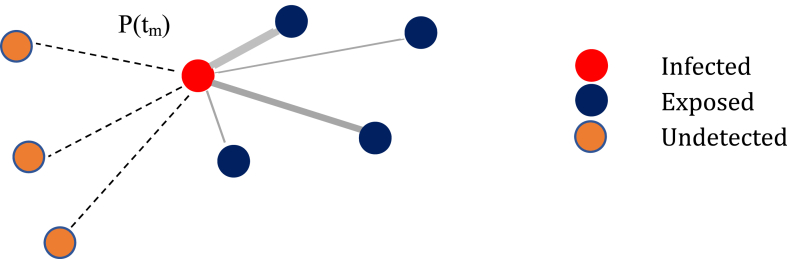
Undetected cases of infection at t_m_ time point.

The analysis of the scenario implies that, based on the record of detected cases of infection in the graph model, the main advantage of such approach is that it allows for determining undetected cases of infection by considering social distancing and the duration of contact (based on mobile application data) and hence, eliminating the uncertainty significantly. The analysis of each confirmed case and investigation of undetected cases of infection and death is paramount from the point of view of obtaining more realistic forecast results. As shown in Fig. 7 for determining the number of undetected cases we use reverse analysis confirmed case by considering social distancing and the contact duration at t_m_ moment. Tracking of a reported case and contacts with the infected person gives the opportunity for a detailed investigation of undetected cases of infection. In this case, of course possible that in the next steps of the spread of infection can be observed that some of the unreported cases at the previous t_m_ moment will discover in one of the compartments or groups (for example, hospitalized or in quarantine at home and will recover). It will give additional information for the decision-making process and confirmation provided actions and eliminating the uncertainty substantially.

Colored graph-based model will enable the assessment and forecasting of detected and undetected cases of recoveries and deaths by considering the dynamics starting from the record of first case of infection, various spatial and chronological characteristics (age, nationality, gender, profession, medical condition etc.).

Currently, the analysis of the statistical data of COVID-19 spread by countries shows that although the human-to-human transmission of the virus is accompanied with spikes as a result of the impact of various factors in some countries, it was possible to stabilize the increase in the number of infections as a result of preventive isolation and quarantine measures in other countries. Surely, the increase in the number of cases of infection is affected by social, economic, demographic factors, the population density, mental values etc. and hence, it is not possible to consider all factors in the study. The future research will review the analysis and consideration of multiple factors affecting the number of infections.

## Conclusion

The governments, non-governmental organizations, experts and epidemiologists attempt to utilize models in order to understand how to respond to, fight and treat the pandemic. The main challenges are the substantial differences between models and the use of different datasets and methodologies in each country. It must particularly be mentioned that the majority of forecasts about COVID-19 is dependent on human behavior, demographic structure and cultural and mental values. In this regard, it is evident that a large number of research questions exist regarding the COVID-19 pandemic and we do not state that we have covered all of them. There is a need for developing realistic models considering the human behavior for the purpose of fighting COVID-19 pandemic at global level and preventing future cases of pandemic.

The article investigates the studies related to the modelling of COVID-19 pandemic. The majority of proposed models emphasizes the major role of direct, human-to-human transmission of COVID-19 infection. On the other hand, despite the existence of large number of proposed models, the majority of them does not take into account the epidemiological characteristics of COVID-19 such as social distancing, duration of contact with a patient as well as important factors affecting the scale of infection. Currently, technologies for monitoring the maintenance of social distance, contact tracing apps adopted by several governments are used as preventive measures among others. It must be noted that the application of these technologies will play an important role in measuring the social distance and determining the risk of infection. The article proposes a conceptual model of COVID-19 epidemic by considering social distance, the duration of contact with the patient and individual information (age, gender, nationality, the presence of chronic diseases and etc.). Considering the epidemiological characteristics of the infection, a graph model of human-to-human transmission of the virus is developed and visualized starting from the detection of first case of infection based on the hypothetical infection scenario. The process of the spread of COVID-19 is reviewed experimentally. The main advantage of the utilization of graph-based model is the possibility of reverse analysis of the spread in the model which allows for determining the number of undetected cases of infection by considering social distancing and the contact duration and hence, eliminating the uncertainty substantially. Notwithstanding, the rapid spread of COVID-19 infection is affected by social, economic factors, population density, mental values and etc. Hence, it was not possible to consider all factors in the study. The analysis of the factors affecting the number of infections will allows for developing more realistic models in future research.

## Declaration of competing interest

We have no conflict of interest to declare.
